# Role of 5-hydroxytryptamine type 3 receptors in aerobic exercise–induced improvement of memory and hippocampal synaptic plasticity

**DOI:** 10.4103/NRR.NRR-D-24-00463

**Published:** 2024-12-07

**Authors:** Xiaoqian He, Ziying Lai, Xueyan Wang, Jingjing Li, Guangbing Duan, Junwen Wang, Zhao Qin, Shuchang Xu, Ying Huang

**Affiliations:** 1 Key Laboratory of Spine and Spinal Cord Injury Repair and Regeneration (Ministry of Education), Department of Physiology and Pharmacology, Tongji Hospital, School of Medicine, Tongji University, Shanghai, China; 2 Department of Gastroenterology, Tongji Institute of Digestive Diseases, Tongji Hospital, School of Medicine, Tongji University, Shanghai, China

**Keywords:** 5-hydroxytryptamine, 5-hydroxytryptamine type 3 receptor, aerobic exercise, brain-derived neurotrophic factor, exploratory memory, hippocampus, long-term potentiation, neurogenesis, neuroglia proliferation, spatial memory

## Abstract

Aerobic exercise facilitates synaptic plasticity, thereby improving cognitive functions such as learning and memory. The 5-hydroxytryptamine system has been indicated in these processes. 5-Hydroxytryptamine type 3 receptors are necessary for exercise-induced hippocampal neurogenesis. Some antipsychotic drugs with 5-hydroxytryptamine type 3 receptor antagonistic properties may impede the amelioration of cognitive impairment and hippocampal plasticity induced by exercise. However, the mechanisms underlying the facilitation of synaptic plasticity by aerobic exercise have not yet been elucidated. In this study, we found that 5-hydroxytryptamine type 3 receptors played an important role in aerobic exercise–mediated improvement of hippocampal-dependent spatial and exploratory memory in mice. While 5-hydroxytryptamine type 3 receptors did not affect baseline neurogenesis in the hippocampal dentate gyrus, 5-hydroxytryptamine type 3 receptors were required for aerobic exercise–induced neurogenesis and astrocyte proliferation in this region. In addition, 5-hydroxytryptamine type 3 receptors were crucial for maintaining long-term potentiation in the CA1, dentate gyrus, and CA3 regions of the hippocampus. The long-term potentiation changes induced by aerobic exercise in sub-regions of the hippocampus were heterogeneous: 5-hydroxytryptamine type 3 receptors were essential for aerobic exercise to enhance long-term potentiation in the CA3, but not the CA1 or dentate gyrus, regions of the hippocampus. Furthermore, aerobic exercise up-regulated 5-hydroxytryptamine type 3 receptor expression and increased brain-derived neurotrophic factor release in the hippocampus in a 5-hydroxytryptamine type 3 receptor–dependent manner. These results suggest that aerobic exercise increases hippocampal dentate gyrus neurogenesis and astrocyte proliferation via the up-regulation of 5-hydroxytryptamine type 3 receptors, leading to more brain-derived neurotrophic factor production and release from these cells, which results in long-term potentiation facilitation in the hippocampal CA3 region and help improve memory. Our findings provide insight into the mechanisms by which physical activity enhances memory and may have implications for improving memory through modulating 5-hydroxytryptamine type 3 receptor.

## Graphical abstract

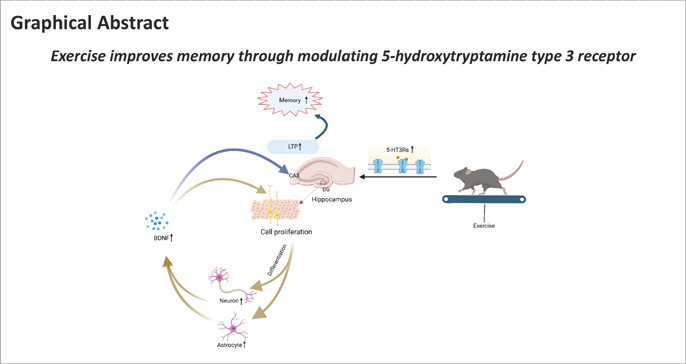

## Introduction

Growing evidence suggests that aerobic exercise improves cognition and reduces cognitive decline, as well as dementia risk (Dominguez et al., 2021; Iso-Markku et al., 2022). Exercise improves cognitive function in children, adults (Yao et al., 2024), adolescents (Polevoy et al., 2022), and the elderly (Xiong et al., 2021). Moreover, studies have shown that exercise attenuates cognitive impairment in patients with Alzheimer’s disease (Li et al., 2024), Parkinson’s disease (Xu et al., 2019), schizophrenia (Roell et al., 2024), and depression (Schuch and Stubbs, 2019). Animal studies also suggest that exercise improves fear conditioning, spatial memory, and exploratory memory in rodents (Nicolas et al., 2024; Xiao et al., 2024; Yu et al., 2024; Zhao et al., 2024). Recent studies suggest that the underlying mechanisms involved in these processes include up-regulating the expression of neurotransmitters and growth factors, inducing neurogenesis, and reducing the expression of inflammatory cytokines to exert neuroprotective effects (Ding et al., 2006; El Hayek et al., 2019; Wang and Han, 2020; Kondo, 2023).

5-Hydroxytryptamine (5-HT), appears early in brain development and plays a critical role in regulating the maturation of neural systems, as well as functioning as a neurotransmitter in adult brains (Brummelte, 2017). In early life, 5-HT helps to shape pathways that contribute to learning, thinking, and stress reactivity. Moreover, 5-HT is associated with adult neurogenesis in the hippocampus (Higuchi and Arakawa, 2023). 5-HT has been extensively studied for its role in psycho-emotional manifestation, as well as cognition. In the hippocampus, the dentate gyrus (DG) is enriched with serotonergic fibers from 5-HT neurons originating in the raphe nucleus (Moore and Halaris, 1975; Oleskevich and Descarries, 1990). Therefore, the 5-HT system regulates the structure and neuronal plasticity of the DG, which is involved in cognition and emotional disorders (Zhao et al., 2008; Ghaheri et al., 2022). Additionally, it has been suggested that exercise increases the concentration of 5-HT in the hippocampus and raphe nuclei (Pietrelli et al., 2018), thereby regulating cognition and memory (Zhang et al., 2020).

The 5-hydroxytryptamine type 3 receptor (5-HT3R), the only ligand-gated cation channel in the 5-HT receptor family, is permeable to Na^+^, Ca^2+^, and K^+^ (Sharp and Barnes, 2020). Evidence suggests that the 5-HT3R plays a role in cognitive function (Huang et al., 2021). A previous clinical study has shown that mutations in 5-HT3Rs are related to cognitive impairment (Thompson et al., 2006). 5-HT3Rs are expressed specifically in cholecystokinin/vasoactive intestinal polypeptide-containing interneurons (INs) in the hippocampus, where they regulate fast synaptic transmissions (Férézou et al., 2002; Du et al., 2024). In addition, the 5-HT3R is essential for exercise-induced hippocampal neurogenesis (Kondo et al., 2015). Antipsychotic medications, which antagonize 5-HT3R function, may therefore impede the promotion of hippocampal plasticity by exercise in patients with schizophrenia undergoing antipsychotic treatment (Rammes et al., 2004; Kim et al., 2018). However, the roles of 5-HT3Rs in exercise-induced memory improvement and the underlying mechanisms remain unclear.

In this study, we used a 5-HT3AR knockout (KO) mouse line to investigate the role and mechanisms of 5-HT3Rs in exercise-induced facilitation of hippocampal synaptic plasticity and memory. Our findings show that exercise facilitates hippocampal long-term potentiation (LTP) and enhances memory by up-regulating 5-HT3R expression in the hippocampus.

## Methods

### Animals

Male C57BL/6 mice and 5-HT3AR knockout mice (5-HT3R KO, The Jackson Laboratory, Bar Harbor, ME, USA, B6.129X1-Htr3atm1jul/J, RRID: IMSR_JAX:005251)) were used in the experiments. Mice were housed at an specific pathogen free animal facility with a 12/12-hour light/dark cycle and *ad libitum* access to food and water. The mice were housed in ventilated cages (five or fewer per cage) with corncob as padding. Cage mates were littermates. The genotypes of the mice were verified by PCR using tail DNA (Aryal and Parker-Thornburg, 2020). The mice (4–6 weeks old) were randomly divided into the following groups: wild-type (WT), wild-type with exercise (WT-E), 5-HT3R KO (KO), and 5-HT3R KO with exercise (KO-E). The mice in the WT-E group and the KO-E group were subjected to an exercise routine for 4 weeks. All experiments were then performed on the 8- to 10-week-old mice. The experiments were approved by Tongji University’s Animal Care and Use Committee (approval No. TJAA07824101). All animal experiments were reported in accordance with the ARRIVE guidelines (Animal Research: Reporting of *In Vivo* Experiments; Percie du Sert et al., 2020).

### Exercise protocol

The mice in the exercise groups used a treadmill for 4 weeks. The exercise training protocol was established on the basis of the Bedford standard (Bedford et al., 1979) and relevant literature (Toy et al., 2014; Wang et al., 2024). Mice in the exercise groups (WT-E group and KO-E group) were allowed a 3-day adaptation period to the treadmill (RWD, LE8710MTS, Shenzhen, China) before the exercise protocol began. During the 3 days of adaptive training, the treadmill speed was set at 10 m/min, with a session duration of 10 minutes for day 1, 20 minutes for day 2, and 30 minutes for day 3. Following the adaptation phase, the mice were subjected to 4 weeks of treadmill exercise with the following protocol: the treadmill speeds in the first, second, third, and fourth weeks were 12, 13, 13, and 14 m/min, respectively. Exercise was performed 6 days a week for 30 minutes every day (**Additional Figures 1** and **2**).

### Brain slice preparation

Mice (8–10 weeks old) were anesthetized with isoﬂurane (0.2 mL isoflurane per mouse, used in a simple drop-chamber system, Sigma, St. Louis, MO, USA, Cat# 792632) before being decapitated. The brain was rapidly removed and immersed in an ice-cold cutting solution that contained MgCl_2_ 4 mM, KCl 2.5 mM, CaCl_2_ 0.5 mM, NaH_2_PO_4_ 1.2 mM, NaHCO_3_ 26 mM, sucrose 252 mM, and glucose 10 mM, saturated with 95% oxygen and 5% carbon dioxide. Coronal hippocampal sections (350 µm) were cut at 4–6°C using a Leica VT1000S vibratome (Leica Instruments, Wetzlar, Germany) in a cutting solution that contained oxygen. The slices were incubated in artificial cerebral spinal fluid (ACSF) (Sinopharm, Beijing, China) saturated with 95% oxygen and 5% carbon dioxide at room temperature for 1 hour before recording. The ACSF contained NaCl 119 mM, MgCl_2_ 2 mM, KCl 2.5 mM, NaH_2_PO_4_ 2.5 mM, NaHCO_3_ 26 mM, CaCl_2_ 2 mM, and glucose 10 mM.

### Electrophysiological recordings

All electrophysiological recordings were conducted using an Axopatch-700B amplifier (Molecular Devices, San Jose, CA, USA) with a sampling rate of 10 kHz and filtered at 5 kHz. Field recording of excitatory postsynaptic potentials (fEPSPs) was performed using glass pipettes (with resistances ranging from 2 to 4 MΩ) filled with ACSF. A bipolar stimulating electrode (FHC, Bowdoin, USA) was placed at the Schaffer collaterals (SCs) for LTP recording in the CA1 region, while the recording electrode was placed in the stratum radiatum of the CA1 region. For LTP recording in the DG region, the stimulating electrode was placed at the perforant pathway (PP) of the DG, and the recording electrode was carefully positioned in the medial aspect of the dentate molecular layer (Colino and Malenka, 1993; Singer et al., 2011). Additionally, a bipolar stimulating electrode was placed at the end of the hippocampal DG region, and the recording electrode was placed in the CA3 area for CA3 LTP recording (Maglione et al., 2019). Data analysis was performed using Clampfit 10.0 software (Molecular Devices, Sunnyvale, CA, USA). For the LTP experiments, fEPSPs were evoked by delivering electrical stimulation once every 30 seconds. A stable baseline fEPSP of 30%–50% of the maximal evoked response was recorded for at least 15 minutes. Theta-burst LTP in the CA1 or CA3 region was induced by eight or four bursts (with each burst having four pulses at 100 Hz) delivered at 5 Hz. High-frequency LTP in the DG region was induced by four trains of 20 pulses at 100 Hz delivered every 15 seconds. To monitor changes in synaptic transmission, responses were recorded for 60 minutes after LTP was induced. On the basis of the slope from 30% to 70% of the rising phase of the fEPSP, synaptic strength was quantified. The magnitude of LTP was calculated as the normalized average slope of the fEPSP in the last 10 minutes of recording. Recordings were only included in the data analysis if the baseline was stable (slope change < 10%).

### Immunohistochemistry

The mice were anesthetized with isoﬂurane and transcardially perfused with PBS followed by 4% paraformaldehyde (PFA) in PBS. The brains were removed and postfixed in 4% PFA at 4°C overnight, then cryoprotected in 15% and 30% sucrose and embedded. Next, coronal sections (40 μm) of the entire hippocampus were prepared with a freezing microtome (RWD Minux FS800). Next, immunohistochemical detection of 5-bromo-2′-deoxyuridine (BrdU) was performed as follows: the sections were incubated in 2 M HCl for 30 minutes at 37°C, then neutralized in 0.1 M sodium tetraborate buffer for 10 minutes. Antigen repair solution (Beyotime, Shanghai, China, Cat# P0090) was then applied for 5 minutes. After blocking with 3% goat serum albumin (Beyotime, Cat# C0265) in 0.3% Triton X-100 for 60 minutes, the sections were incubated with primary antibodies at 4°C overnight, followed by incubation with Alexa-labeled secondary antibodies for 1 hour at room temperature. Hippocampal slices from each animal were divided into groups of 10. One slice was selected from each group, and the number of BrdU-positive cells was counted. The total number of BrdU-positive cells was calculated by multiplying the number of those observed in the representative section by 10. A Nikon laser confocal microscope (Nikon, Tokyo, Japan, A1R) was used for image acquisition. The following primary antibodies were used: anti-BrdU (mouse, Sigma, Cat# SAB4700630), anti-5-HT3AR (rabbit, 1:50, Abcam, Cambridge, MA, USA, Cat# ab271031), and anti-neuronal nuclei (anti-NeuN; rabbit, 1:1000, Merk Millipore, Burlington, MA, USA, Cat# ABN90). The following secondary antibodies were used: anti-mouse IgG (H+L) Alexa Fluor(R) 488 (1:1000, Cell Signaling Technology, Boston, MA, USA, Cat# 44125) and anti-rabbit IgG Fab2 Alexa Fluor(R) 555 (1:1000, Cell Signaling Technology, Cat# 44135).

### Morris water maze

In Morris water maze (MWM) test (Aguado et al., 2024), mice were trained for 6 days (with four trials per day) to locate the hidden platform after freely swimming with no cues or platform for a day. In the experiments performed to examine the influence of exercise, to avoid overtraining the mice, we adjusted the training protocol to two trials per day over 4 days. Mice were placed on the water surface in the designated starting position in the maze, facing the tank wall, and released into the water (not dropped). A timer was started when the animal was released and was stopped once the mouse reached the platform. The training time was 1 minute. Animals that did not find the platform in 1 minute were guided to it. On the next day, after 4 or 6 days of training, 1-minute probe tests were conducted in which the mice were released into the maze without platforms. A video tracking system (VanBi, Shanghai, China) was used to monitor all trials. The escape latencies during the training days and the number of times that the mice crossed the former location of the platform during the probe test were recorded and compared.

### Novel object recognition

The novel object recognition (NOR) test was performed in three sessions, as described previously (Antunes and Biala, 2012), with minor modifications. (1) Habituation: In the absence of objects, mice were allowed to explore the open-field arena for five minutes. Then, they were moved out of the arena and placed in the holding cage. The process was repeated three times for 3 days before the test. (2) Familiarization: Each mouse was given 10 minutes to explore two identical objects (A+A) in an open-field arena. Mice were released against the center of the opposite wall with their backs to the objects to prevent them from being coerced into exploring the objects. (3) Test phase: After a retention interval of 1 hour, objects familiar to the mice were replaced with novel ones in the open-field arena, and the mice were allowed a 5-minute exploration period. Computerized image analysis software (VanBi) was used to analyze the paths taken by the animals and the amount of time that they spent exploring each object. The relative discrimination index (DI) was calculated as follows: DI = (exploring time for novel object – exploring time for the familiar object)/total exploring time for objects.

### Quantitative reverse transcription-polymerase chain reaction

The mice were anesthetized, their brains were removed, and mRNA was extracted from the hippocampal tissues for quantitative reverse transcription-polymerase chain reaction (qRT-PCR) assay. cDNA was synthesized using PrimeScript™ RT Master Mix (TaKaRa, Tokyo, Japan). Target gene expression was analyzed using qPCR Master Mix (Wuhan Servicebio Technology, Wuhan, China, G3326) and a QuantStudio7 Flex system (Thermo Fisher Scientific, Waltham, MA, USA, Quanstudio 7 Flex). The qRT-PCR conditions were as follows: denaturation at 95°C for 30 seconds, followed by 40 cycles of 95°C for 15 seconds and 60°C for 30 seconds. mRNA levels were calculated using the ΔΔCt method and normalized to glyceraldehyde-3-phosphate dehydrogenase (GAPDH) expression levels. The following primers were used: *GAPDH* (F: 5′-GTC CTC CTC AAC GTA ACC CA-3′; R: 5′-GGC TGC CAG TTT CAA CAT CA-3′); *Htr3a* (F: 5′-AGG CAC CTG GTC CTA GAC AGA ATA G -3′; R: 5′-TCA TCA GTC TTG TTG GCT TGG AAG G-3′).

### Western blotting

Hippocampal tissues from each group were collected and homogenized, total protein was extracted, and the protein concentration was measured by bicinchoninic acid (BCA) assay (Goldring, 2019). Ten micrograms of protein from each sample were subjected to electrophoresis and transferred to PVDF membranes (Millipore, Bedford, MA, USA, Cat# IPVH00010). The membranes were blocked with 5% non-fat dry milk for 1 hour at room temperature. Next, the membranes were incubated overnight at 4°C with the following primary antibodies: anti–postsynaptic density 95 (PSD95; rabbit polyclonal to PSD95, 1:1500, Cell Signaling Technology, Cat# 25075), anti-BDNF (rabbit polyclonal to BDNF, 1:1000, Abcam, Cat# ab108319), or anti-synaptophysin (SYP; rabbit polyclonal to SYP, 1:2000, Cell Signaling Technology, Cat# 364065). After washing the membranes, a goat anti-rabbit IgG (1:5000, Beyotime, Cat# A0208) or mouse monoclonal antibody to β-tubulin (1:5000, Cell Signaling Technology, Cat# 2128S) secondary antibody was added to the membranes, which were then incubated for 2 hours at room temperature. Finally, the membranes were treated with a developing reagent (Millipore), and the protein signals were imaged via chemiluminescence (GE Healthcare, Chicago, IL, USA). PSD95 and SYP expression levels were normalized to β-tubulin. BDNF expression was normalized to GAPDH.

### Transmission electron microscopy

The hippocampal tissue was cut into 1 × 1 × 1 mm^3^ pieces, which were then fixed with 1% OsO_4_ in 0.1 M PB (pH 7.4), followed by gradient dehydration with alcohol at room temperature. Next, the pieces were embedded overnight in EMBed 812 (SPIbio, Shanghai, China, 90529-77-4), then polymerized at 60°C for 48 hours. Ultrathin 80-nm sections were taken, mounted on 150-mesh cuprum grids with formvar film, and stained with 2% uranium acetate saturated alcohol solution (SPIbio, 02624-AB) for 8 minutes. The tissues were then rinsed in 70% ethanol three times and ultrapure water three times. Next, the sections were stained with 2.6% lead citrate (Sinopharm, 10019408) for 8 minutes under CO_2_-free conditions and rinsed three times with ultrapure water. The cuprum grids were placed into the grid board, blotted with filter paper, and dried overnight at room temperature. Images of the cuprum grids were taken under TEM (Hitachi, Tokyo, Japan, HT7800/HT7700).

### 5-HT and 5HIAA measurement by high-pressure liquid chromatography

Mouse hippocampal tissues were homogenized at low temperatures. 5-HT and 5HIAA levels were assessed using an Agilent 1200 series neurotransmitter analyzer (Agilent Technologies, Santa Clara, CA, USA). Separations were performed at a flow rate of 0.2 mL/min using a mobile phase of PB containing 0.05 mM EDTA, 1.7 mM orthosilicic acid, 90.0 mM Na_2_HPO_4_, 50.0 mM citric acid, and 5% acetonitrile, with the detector voltage setting at +700 mV. The columns and detector cells were kept at 35°C in a column oven. The data were collected and analyzed by ChemStation (Agilent Technologies).

### Statistical analysis

Statistical analysis was conducted using GraphPad Prism (version 9.4.1, GraphPad Software, San Diego, CA, USA, www.graphpad.com). Data are presented as mean ± standard error of the mean (SEM). Unpaired *t*-test was used to analyze data from the Morris water maze test, hippocampal field potential recordings, RT-PCR, immunofluorescence assay and high-pressure liquid chromatography. One-way analysis of variance followed by Tukey’s *post hoc* test or Kruskal–Wallis test was used to analyze data from the Novel object recognition, immunofluorescence assay, western blotting, TEM images. *P* < 0.05 was considered statistically significant.

## Results

### 5-Hydroxytryptamine type 3 receptors are required for exercise-induced memory improvement

**Figure 1:**
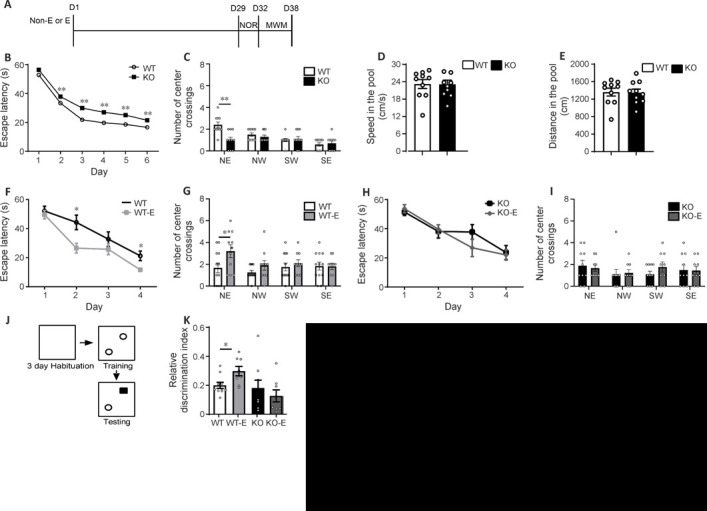
5-HT3Rs are required for exercise-induced memory improvement. (A) Timeline of the behavioral experiments. (B) Latency for finding the platform in the probe phase of the Morris water maze tests for WT and KO mice (WT, *n* = 10; KO, *n* = 10; 4 trails/day). (C) Number of crossings of the center (location of the platform) in the training quadrant *versus* the non-trained quadrants (WT, *n* = 10; KO, *n* = 10) in the probe test (NE: northeast, NW: northwest, SE: southeast, SW: southwest). Swimming speed (D) and distance (E) in the water maze (WT, *n* = 10; KO, *n* = 10). (F) Latency for finding the platform in the acquisition phase in WT and WT-E mice (WT, *n* = 10; WT-E, *n* = 10) in the Morris water maze tests (2 trails/day). (G) Number of crossings of the center (location of the platform) of the training quadrant versus the non-trained quadrants (WT, *n* = 10; WT-E, *n* = 10) in probe test. (H) Latency for finding the platform in the acquisition phase in KO and KO-E mice (KO, *n* = 10; KO-E, *n* = 9; 2 trails/day). (I) Number of crossings of the center (location of the platform) of the training quadrant *versus* the non-trained quadrants (KO, *n* = 10; KO-E, *n* = 9) in probe test. (J) New object recognition experiment diagram. (K) Discrimination index in the new object recognition experiment (WT, *n* = 10; KO, *n* =10; WT-E, *n* =10; KO-E, *n* = 9). *P < 0.05, **P < 0.01. Data are shown as the mean ± SEM. 5-HT3R: 5-Hydroxytryptamine type 3 receptor; KO: knockout; WT: wild-type.

To investigate the impact of exercise on memory and the involvement of 5-HT3Rs in this process, we first performed behavioral tests on mice in the four groups (WT, WT-E, KO, and KO-E). MWM tests and NOR tests were used to examine spatial memory and exploratory memory (**Figure 1A**).

In the MWM tests, during 6 days of training, the escape latency of mice in the KO group was longer than that of mice in the WT group (without exercise, *P* < 0.01; **Figure 1B**). In the probe test, mice in the WT group crossed the target quadrant platform more frequently than those in the KO group (*P* < 0.01; **Figure 1C**). There was no significant difference in swimming distance or speed between mice in the KO and WT groups (*P* > 0.05; **Figure 1D** and **E**). More importantly, the escape latency of mice in the WT-E group was significantly shorter than that of mice in the WT group (*P* < 0.05 for day 2 and day 4; **Figure 1F**). In the probe test, mice in the WT-E group crossed the target quadrant platform more frequently than mice in the WT group (*P* < 0.05; **Figure 1G**). However, there was no difference in escape latency or the number of times crossing the target quadrant platform between mice in the KO and KO-E groups (*P* > 0.05; **Figure 1H** and **I**).

In the NOR tests, there was no significant difference in exploration time of new objects between the WT and KO groups (without exercise, *P* > 0.05; **Figure 1J** and **K**). However, mice in the WT-E group exhibited longer exploration time of new objects (*P* < 0.05; **Figure 1K**). There was no difference in exploration time of new objects between the KO and KO-E groups (*P* > 0.05; **Figure 1K**).

These results suggest that the 5-HT3R is required for exercise-mediated improvement of hippocampal-dependent spatial memory and explorative memory in mice.

### Exercise promotes hippocampal neurogenesis and astrocyte proliferation through 5-hydroxytryptamine type 3 receptors

**Figure 2:**
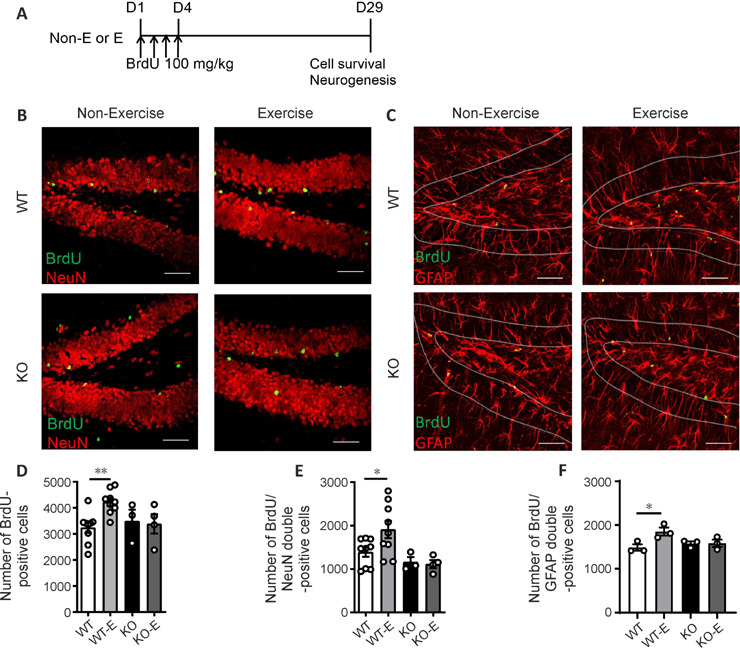
Exercise facilitates neurogenesis and astrocyte proliferation through 5-HT3Rs in the hippocampal dentate gyrus. (A) Timeline of the cell proliferation and neurogenesis experiments (see detailed description in the Methods section). (B) Representative images of the hippocampal dentate gyrus, double-positive for BrdU and NeuN. Data for WT, KO, WT-E, and KO-E mice are shown. Scale bars: 50 μm. (C) Representative images of the hippocampal dentate gyrus, double-positive for BrdU and GFAP. Data for WT, KO, WT-E, and KO-E mice are shown. Scale bars: 50 μm. (D) Number of BrdU-positive cells (WT, *n* = 9; WT-E, *n* = 9; KO, *n* = 3; KO-E, *n* = 4). (E) Number of the BrdU/NeuN double-positive cells (WT, *n* = 9; WT-E, *n* = 9; KO, *n* = 3; KO-E, *n* = 4). (F) Number of BrdU/GFAP double-positive cells (WT, *n* = 3; WT-E, *n* = 3; KO, *n* = 3; KO-E, *n* = 3). *P < 0.05, **P < 0.01. Data are shown as the mean ± SEM. 5-HT3R: 5-Hydroxytryptamine type 3 receptor; BrdU: bromodeoxyuridine; E: exercise; GFAP: glial fibrillary acidic protein; NeuN: neuronal nuclei; KO: knockout; WT: wild-type.

We examined the effect of exercise on hippocampal cell proliferation and the possible role of 5-HT3Rs in this process by staining brain sections with BrdU to label newborn cells and NeuN to label all neurons. There was no significant difference in the numbers of BrdU-positive (proliferating) and BrdU/NeuN double-positive (newly formed neurons) cells between WT and KO mice (*P* > 0.05; **Figure 2A**, **B**, **D**, and **E**). Compared with the WT group, hippocampal DG cell proliferation and the number of newborn neurons were significantly greater in the WT-E group (number of BrdU-positive cells: WT *versus* WT-E, *P* < 0.01; number of BrdU/NeuN double-positive cells: WT *versus* WT-E, *P* < 0.05; **Figure 2D** and **E**). However, there was no difference in the numbers of single-or double-labeled cells between the KO and KO-E groups (*P* > 0.05; **Figure 2D** and **E**). Additionally, GFAP labeling showed that there was a significant increase in the number of newborn astrocytes in the WT-E group compared with the WT group (number of BrdU/GFAP double-positive cells: WT *versus* WT-E, *P* < 0.05; **Figure 2C** and **F**). However, no significant difference in the number of newborn astrocytes was found between the KO and KO-E groups (*P* > 0.05; **Figure 2C** and **F**). These results indicate that the 5-HT3R is not required for baseline cell survival and neurogenesis in the hippocampal DG, but is necessary for exercise-induced cell proliferation, including neurogenesis and astrocyte regeneration, in the hippocampal DG.

### Exercise enhances synaptic plasticity in the hippocampal CA3 region through 5-hydroxytryptamine type 3 receptors

**Figure 3:**
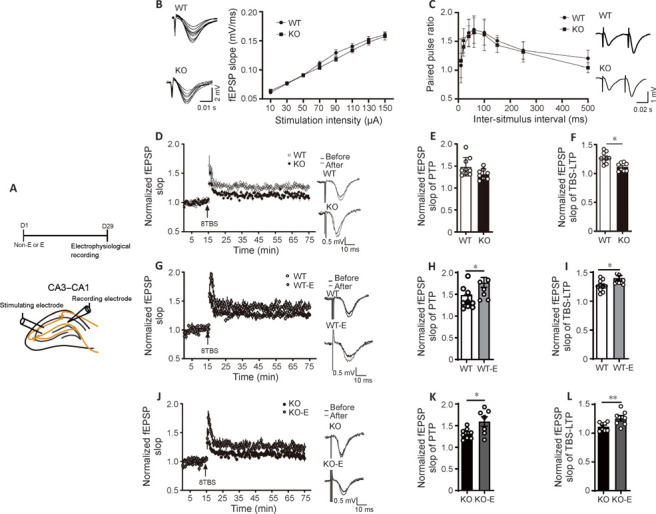
Exercise facilitates hippocampal CA1 synaptic plasticity in a 5-HT3R-independent manner. (A) Timeline and schematic diagram of hippocampal CA1 field potential recording. (B) Input-output curve and (C) paired-pulse ratios of the CA1 fEPSP. (D) Theta-burst stimulation (TBS)-induced CA3-CA1 LTP in hippocampal sections from WT and KO mice. Inset in D: typical fEPSP recordings taken before TBS (black) or 60 minutes after LTP induction (gray) (WT, *n* = 9; KO, *n* = 9). (E) Changes in PTP after stimulation (WT, *n* = 9; KO, *n* = 9). (F) LTP magnitude (WT, *n* = 9; KO, *n* = 9). (G) TBS-induced CA3–CA1 LTP in hippocampal sections from WT and WT-E mice (WT, *n* = 9; WT-E, *n* = 8). (H) Changes in PTP after stimulation (WT, *n* = 9; WT-E, *n* = 8). (I) LTP magnitude (WT, *n* =9; WT-E, *n* =8). (J) TBS-induced CA3-CA1 LTP in hippocampal sections from KO and KO-E mice. (KO, *n* = 9; KO-E, *n* = 9). (K) Changes in PTP after stimulation (KO, *n* = 9; KO-E, *n* = 9). (L) LTP magnitude (KO, *n* = 9; KO-E, *n* = 9). *P < 0.05, **P < 0.01. Data are shown as the mean ± SEM. 5-HT3R: 5-Hydroxytryptamine type 3 receptor; E: exercise; fEPSP: ﬁeld excitatory postsynaptic potential; KO: knockout; PTP: post-tetanic potentiation; WT: wild type.

To further investigate the effect of exercise on hippocampal synaptic transmission and the role of 5-HT3Rs in this process, we compared EPSPs in the hippocampus among the four groups of mice. Because CA1 is the region in the hippocampus that projects output signals, we first recorded fEPSPs in the CA1 region of hippocampal sections in response to stimulation of the SC pathway (**Figure 3A**). There was no difference between WT and KO mice in input/output relation or paired-pulse ratio (PPR) of fEPSP slopes (*P* > 0.05; **Figure 3B** and **C**), suggesting that CA1 basal synaptic transmission is unaffected by 5-HT3R disruption. Additionally, compared with the WT group, there was no difference in post-tetanic potentiation (PTP) in the KO group (*P* > 0.05; **Figure 3D** and **E**), while the LTP in the CA1 region of the KO group was impaired (WT *versus* KO, *P* < 0.05; **Figure 3D** and **F**). The PTP and LTP of the CA1 region were significantly enhanced in the WT-E group compared with the WT group (PTP: WT *versus* WT-E, *P* < 0.05; **Figure 3G** and **H**; LTP: WT *versus* WT-E, *P* < 0.05; **Figure 3G** and **I**). Moreover, the PTP and LTP of the CA1 region were significantly enhanced in the KO-E group compared with the KO group (PTP: KO *versus* KO-E, *P* < 0.05; **Figure 3J** and **K**, LTP: KO *versus* KO-E, *P* < 0.01; **Figure 3J** and **L**). These results suggest that exercise-induced enhancement of synaptic plasticity in the hippocampal CA1 region is not dependent on 5-HT3Rs.

**Figure 4:**
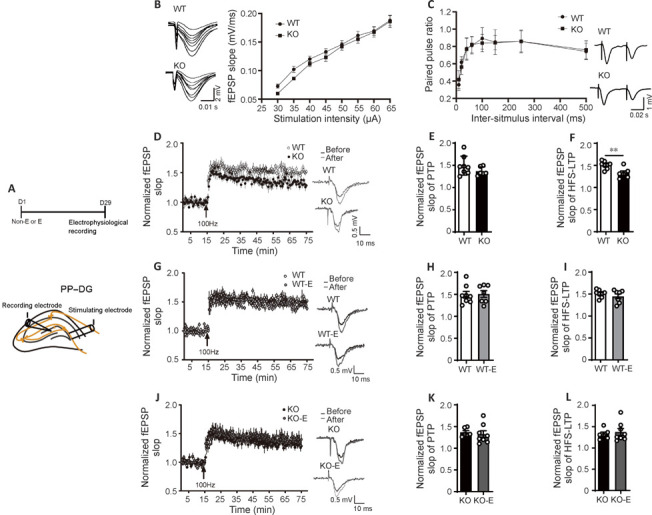
Exercise does not alter LTP in the hippocampal DG region. (A) Timeline and schematic diagram of hippocampal DG field potential recording. (B) Input–output curve and (C) paired-pulse ratios of the DG fEPSP. (D) HFS-induced PP-DG LTP in hippocampal sections from WT and KO mice. Inset in D: typical fEPSPs taken before HFS (black) or 60 minutes after LTP induction (gray) (WT, *n* = 8; KO, *n* = 6). (E) Changes in PTP after stimulation (WT, *n* = 8; KO, *n* = 6). (F) LTP magnitude (WT, *n* = 8; KO, *n* = 6). (G) HFS-induced PP-DG LTP in hippocampal sections from WT and WT-E mice (WT, *n* = 8; WT-E, *n* = 7). (H) Changes in PTP after stimulation (WT, *n* = 8; WT-E, *n* = 7). (I) LTP magnitude (WT, *n* = 8; WT-E, *n* = 7). (J) HFS-induced PP-DG LTP in hippocampal sections from KO and KO-E mice (KO, *n* = 6; KO-E, *n* = 8). (K) Changes in PTP after stimulation (KO, *n* = 6; KO-E, *n* = 8). (L) LTP magnitude (KO, *n* = 6; KO-E, *n* = 8). **P < 0.01. Data are shown as the mean ± SEM. 5-HT3R: 5-Hydroxytryptamine type 3 receptor; DG: dentate gyrus; E: exercise; fEPSP: field recording of excitatory postsynaptic potential; HFS: high-frequency stimulation; KO: knockout; LTP: long-term potentiation; PP: perforant pathway; WT: wild-type.

Given our finding that exercise promotes neurogenesis and astrocyte regeneration in the hippocampal DG through 5-HT3Rs, next we recorded synaptic transmission in the DG region of the hippocampus in response to direct PP stimulation (**Figure 4A**). There was no difference between WT and KO mice in terms of input/output relation or PPR of the fEPSP slopes (*P* > 0.05; **Figure 4B** and **C**), suggesting that basal synaptic transmission in the DG region was unaffected by 5-HT3R disruption. Furthermore, there was no significant change in the PTP in the hippocampal DG region in the KO group compared with the WT group (*P* > 0.05; **Figure 4D** and **E**), while the LTP in the DG region of the KO group was impaired (WT *versus* KO, *P* < 0.01; **Figure 4D** and **F**). In addition, there was no significant change in PTP and LTP in the WT-E group compared with the WT group (*P* > 0.05; **Figure 4G–I**). There was also no change PTP or LTP in the DG region between the KO and KO-E groups (*P* > 0.05; **Figure 4J–L**), suggesting that exercise does not improve synaptic plasticity in the hippocampal DG region.

**Figure 5:**
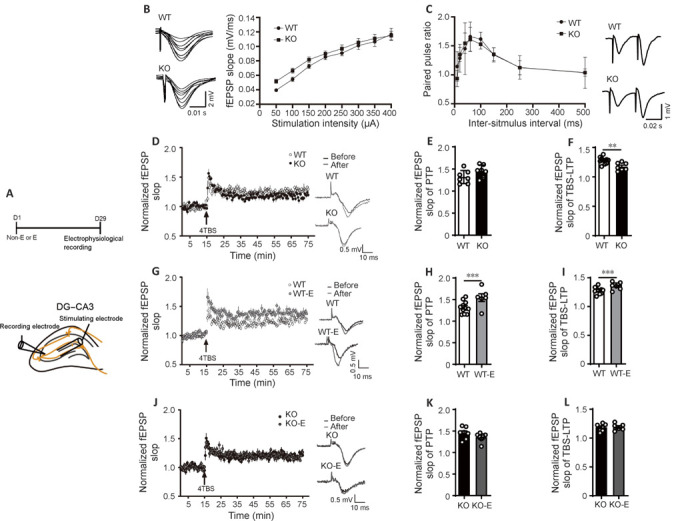
Exercise enhances synaptic plasticity in the hippocampal CA3 region through 5-HT3Rs. (A) Timeline and schematic diagram of hippocampal CA3 field potential recording. (B) Input–output curve and (C) paired-pulse ratios of the CA3 fEPSP. (D) TBS-induced DG-CA3 LTP in hippocampal sections from WT and KO mice. Inset in D: typical fEPSPs taken before TBS (black) or 60 minutes after LTP induction (gray) (WT, *n* = 11; KO, *n* = 7). (E) Changes in PTP after stimulation (WT, *n* = 11; KO, *n* = 7). (F) LTP magnitude (WT, *n* = 11; KO, *n* = 7). (G) TBS-induced DG-CA3 LTP in hippocampal sections from WT and WT-E mice (WT, *n* = 11; WT-E, *n* = 7). (H) Changes in PTP after stimulation (WT, *n* = 11; WT-E, *n* = 7). (I) LTP magnitude (WT, *n* = 11; WT-E, *n* = 7). (J) TBS-induced DG-CA3 LTP in hippocampal sections from KO and KO-E mice (KO, *n* = 7; KO-E, *n* = 6). (K) Changes in PTP after stimulation (KO, *n* =7; KO-E, *n* = 6). (L) LTP magnitude (KO, *n* = 7; KO-E, *n* = 6). **P < 0.01, ***P < 0.001. Data are shown as the mean ± SEM. 5-HT3R: 5-Hydroxytryptamine type 3 receptor; DG: dentate gyrus; E: exercise; fEPSP: field recording of excitatory postsynaptic potential; KO: knockout; WT: wild-type.

Electrophysiological recordings of the hippocampal CA3 region (which receives projections from the DG), in response to stimulation of mossy fiber pathway (MF) (**Figure 5A**), showed no difference between WT and KO mice in terms of input/output relation and PPR of the fEPSP slopes (*P* > 0.05; **Figure 5B** and **C**), suggesting that basal synaptic transmission in the CA3 region is unaffected by 5-HT3R disruption. Compared with the WT group, there was no significant difference in PTP in the CA3 region of the KO group (*P* > 0.05; **Figure 5D** and **E**), but the LTP was impaired in the KO group (WT *versus* KO, *P* < 0.05; **Figure 5D** and **F**). Additionally, the PTP and LTP in the CA3 region were significantly higher in the WT-E group than in the WT group (PTP: WT *versus* WT-E, *P* < 0.05; **Figure 5G** and **H**, LTP: WT *versus* WT-E, *P* < 0.05; **Figure 5G** and **I**). However, there was no difference in the PTP and LTP in the CA3 region between the KO and KO-E groups (*P* > 0.05; **Figure 5J–L**). These results suggest that 5-HT3Rs are required for exercise-mediated enhancement of LTP in the CA3 region of the hippocampus.

### 5-Hydroxytryptamine type-3 receptors are required for the exercise-induced increase in hippocampal BDNF expression

**Figure 6:**
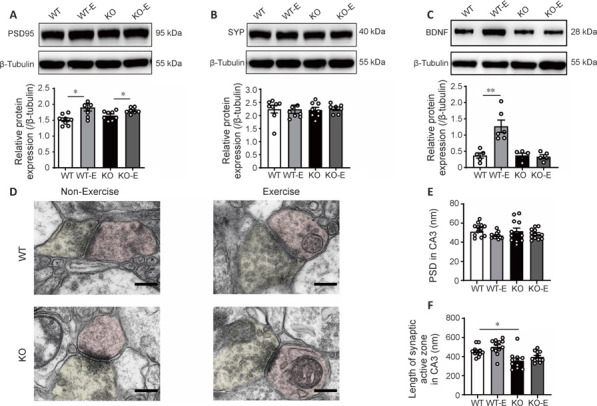
5-HT3Rs are required for the exercise-induced increase in hippocampal BDNF expression. (A, B) PSD95 (A) and SYP (B) protein expression levels in the hippocampus (WT, *n* = 8; WT-E, *n* = 8; KO, *n* = 8; KO-E, *n* = 7). (C) BDNF protein expression levels in the hippocampus (WT, *n* = 5; WT-E, *n* = 6; KO, *n* = 5; KO-E, *n* = 5). (D) Transmission electron microscopy images of synapses in the CA3 region of the hippocampus. Scale bars: 200 nm (yellow: presynaptic, red: postsynaptic terminals). (E, F) PSD (E) and length of active zone (F) (WT, *n* = 3 mice [12 sections]; KO, *n* = 3 mice [12 sections]; WT-E, *n* = 3 mice [12 sections]; KO-E, *n* = 3 mice [12 sections]). *P < 0.05; **P < 0.01. Data are shown as the mean ± SEM. 5-HT3R: 5-Hydroxytryptamine type 3 receptor; BDNF: brain-derived neurotrophic factor; E: exercise; KO: knockout; PSD95: postsynaptic density 95; SYP: synaptophysin; WT: wild-type.

To study the effects of exercise on hippocampal synaptic plasticity-related protein expression and the role of 5-HT3Rs in this process, we assessed expression levels of the synaptic plasticity–related protein PSD95. There was no significant difference in PSD95 levels in the WT group compared with the KO group (*P* > 0.05; **Figure 6A**); however, PSD95 levels increased significantly in the WT-E group compared with the WT group (WT *versus* WT-E, *P* < 0.05; **Figure 6A**). PSD95 levels were significantly increased in the KO-E group compared with the KO group (KO *versus* KO-E, *P* < 0.01; **Figure 6A**). Moreover, there was no significant difference in SYP levels between the WT and KO groups (*P* > 0.05; **Figure 6B**) or between the WT and WT-E groups (*P* > 0.05; **Figure 6B**). Next, we examined the levels of BDNF, which have been found to facilitate synaptic plasticity in the hippocampus. Western blotting showed no significant difference in BDNF levels between the WT and KO groups (*P* > 0.05; **Figure 6C**). BDNF levels in the WT-E group were significantly increased compared with the WT group (WT *versus* WT-E, *P* < 0.01; **Figure 6C**). However, there was no significant difference in BDNF levels between the KO and KO-E groups (*P* > 0.05; **Figure 6C**). These results indicate that exercise up-regulates PSD95 and BDNF expression and that 5-HT3Rs play an important role in the exercise-induced increase in BDNF levels.

Next, we investigated the synaptic structure of the CA3 region of the hippocampus by electron microscopy and found that the synaptic active zone was shorter in the KO group than in the WT group (WT *versus* KO, *P* < 0.05; **Figure 6D** and **F**). There was no difference in postsynaptic density between the WT and WT-E groups or between the KO and KO-E groups (*P* > 0.05; **Figure 6D** and **E**).

### Exercise promotes 5-hydroxytryptamine type-3 receptor expression in the hippocampus

**Figure 7:**
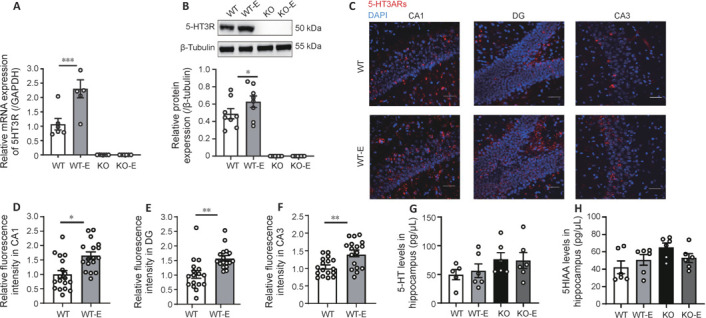
Exercise promotes 5-HT3R expression in the hippocampus. (A) 5HT3R mRNA expression in the hippocampus (WT, *n* = 6; WT-E, *n* = 5; KO, *n* = 6; KO-E, *n* = 6). (B) 5HT3R protein expression in the hippocampus (WT, *n* = 8; WT-E, *n* = 8; KO, *n* = 8; KO-E, *n* = 8). 5-HT3AR immunostaining (C) and quantitative analysis of 5-HT3R expression in the CA1 (D), DG (E), and CA3 (F) regions of the hippocampus. Scale bars: 25 μm (*n* = 18 sections from three mice). (G) 5-HT levels in the hippocampus (WT, *n* = 5; WT-E, *n* = 6; KO, *n* = 6; KO-E, *n* = 6). (H) 5HIAA levels in the hippocampus (WT, *n* = 6; WT-E, *n* = 6; KO, *n* = 6; KO-E, *n* = 6). *P < 0.05; **P < 0.01, ***P < 0.001. Data are shown as the mean ± SEM. 5-HT: 5-Hydroxytryptamine; 5HIAA: 5-hydroxyindoleacetic acid; 5-HT3R: 5-hydroxytryptamine type 3 receptor; DG: dentate gyrus; E: exercise; KO: knockout; PSD95: postsynaptic density 95; SYP: synaptophysin; WT: wild-type.

To further assess the effects of exercise on 5-HT3R expression, we examined 5-HT3R mRNA expression levels in the hippocampus. The qRT-PCR results showed that the level of 5-HT3R mRNA expression was significantly higher in the WT-E group than in the WT group (WT *versus* WT-E, *P* < 0.001; **Figure 7A**), whereas no 5-HT3R mRNA expression was detected in the KO and KO-E groups (*P* > 0.05; **Figure 7A**). Furthermore, western blotting showed that 5-HT3R protein levels were higher in the WT-E group compared with the WT group (WT *versus* WT-E, *P* < 0.05; **Figure 7B**), whereas no 5-HT3R expression was detected in the KO and KO-E groups, and there was therefore no significant difference between these groups (*P* > 0.05; **Figure 7A** and **B**).

To determine whether exercise increased 5-HT3R expression in specific subregions of the hippocampus, we examined exercise-induced changes in 5-HT3R expression in DG, CA3, and CA1 by immunofluorescence (**Figure 7C**). Compared with the WT group, the integral fluorescence intensity of 5-HT3R was significantly increased in all three hippocampal subregions in the WT-E group (CA1, WT *versus* WT-E, *P* < 0.05, **Figure 7D**; DG, WT *versus* WT-E, *P* < 0.01; **Figure 7E**; CA3, WT *versus* WT-E, *P* < 0.01; **Figure 7F**).

Next, to determine whether exercise alters 5-HT release in the hippocampus, we measured the concentration of 5-HT and its metabolite 5HIAA in hippocampal tissue by high-pressure liquid chromatography. The results showed no difference in 5-HT and 5HIAA levels between the WT and WT-E groups (*P* > 0.05; **Figure 7G** and **H**). Similarly, there was no difference in 5-HT and 5HIAA levels between the KO and KO-E groups (*P* > 0.05; **Figure 7G** and **H**). These results suggest that exercise up-regulates 5-HT3R expression.

## Discussion

In this study, we used 5-HT3R KO mice to provide direct evidence that exercise facilitates hippocampal LTP and memory by up-regulating 5-HT3R expression to enhance neurogenesis, astrocyte proliferation, and BDNF release in the hippocampus. We found that 5-HT3Rs were required for exercise-mediated promotion of hippocampus-dependent spatial and exploratory memory in mice. In addition, we showed that 5-HT3Rs played crucial roles in exercise-induced neurogenesis and astrocyte proliferation in the DG region of the hippocampus. Intriguingly, 5-HT3Rs were essential for exercise-induced enhancement of LTP in the CA3, but not in the CA1 or DG. Furthermore, exercise up-regulated 5-HT3R expression and increased hippocampal BDNF release in a 5-HT3R-dependent manner.

The 5-HT system has been suggested to play a critical role in mediating the beneficial effects of exercise on the brain (Kondo et al., 2015). To date, 14 subtypes of 5-HT receptors have been identified by pharmacological analyses and complementary DNA cloning (Sharp and Barnes, 2020). However, which 5-HT receptors mediate these exercise-induced neuronal effects remains unclear. Previous studies indicate that the 5-HT3Rs play important roles in mood and memory (Hassell and Maswood, 2020; Sharp and Barnes, 2020; Bhatt et al., 2021). Additionally, Kondo et al. (2015) reported that 5-HT3Rs are essential for the antidepressant effects of running-wheel exercise, but not for learning and memory enhancement. In contrast, in the current study, we found that 5-HT3Rs are required for aerobic exercise (using a treadmill) to improve hippocampal plasticity, as well as spatial and explorative memory, in mice. This apparent discrepancy may be because of the different exercise patterns and intensities used in the two studies.

Controversy exists regarding the existence and extent of adult hippocampal neurogenesis. A previous study suggested that neurogenesis sharply decreased post-birth and is negligible in adults (Sorrells et al., 2018). However, another study reported lifelong neurogenesis in the DG of healthy humans (Abbott and Nigussie, 2020). Neural stem cells persist in the adult mammalian nervous system (Urbán et al., 2019), and ongoing production, maturation, and integration of these cells into the hippocampal circuit are essential for learning and memory, as evidenced by anatomical and electrophysiological studies (Cameron and Glover, 2015). The findings from our study support the concept of ongoing neurogenesis in the hippocampal DG in young mice. Additionally, extensive research has confirmed that exercise enhances neurogenesis and cognition (Speisman et al., 2013; Hong et al., 2020). 5-HT expression levels parallel the beneficial effects of exercise on neurogenesis in the adult DG (Micheli et al., 2018). In addition, exercise elevates 5-HT expression in the mouse hippocampus (Zhang et al., 2020), and treatment with fluoxetine accelerates neuronal maturation (Wang et al., 2008). In the present study, we showed that treadmill exercise enhanced neurogenesis in the DG of adult mice in a 5-HT3R-dependent manner. Moreover, we found that treadmill exercise also facilitated astrocyte proliferation in the DG via 5-HT3Rs, possibly through an increase in BDNF release, as well as the interaction between 5-HT3R-containing GABAergic interneurons and astrocytes.

Interestingly, we observed notable heterogeneity in LTP changes induced by exercise in different subregions of the hippocampus. Given that exercise increased 5-HT3R expression in all three hippocampal subregions, this suggests that the heterogeneity in LTP changes induced by exercise in different subregions of the hippocampus may be related to where neurogenesis occurs in the hippocampus. The DG is the primary hippocampal region known to exhibit adult neurogenesis. Exercise-induced DG neurogenesis promotes BDNF release from neurons in the DG (Quesseveur et al., 2013; Kraemer and Kraemer, 2023). BDNF can then be transported in an anterograde fashion and released from axon terminals in the CA3 to provide trophic support to CA3 pyramidal neurons (Smith et al., 1997) and facilitate synaptic plasticity in the CA3. Because exercise-induced DG neurogenesis was dependent on 5-HT3Rs, it is not surprising that the LTP enhancement in the CA3 caused by the neurogenesis and increased BDNF release that we observed was 5-HT3R-dependent. Furthermore, exercise may also increase cerebral blood circulation, providing more oxygen to neurons, thereby reducing inflammation and oxidative stress and facilitating synaptic plasticity. The exercise-induced LTP enhancement mediated by these mechanisms could be independent of neurogenesis or 5-HT3Rs, just as we found in the CA1 region in the present study. Another possible reason for the interesting heterogeneity of LTP changes in subregions of the hippocampus may be related to variations in serotonergic fiber distribution or 5-HT3R expression in the hippocampus. One study showed that the CA3 region has the highest density of serotonergic axons, followed by the DG, and that the CA1 region has the lowest density (Mamounas et al., 1991). Nevertheless, further investigation is needed to elucidate the detailed mechanisms of the observations made in the current study.

Previous studies have shown an increase in brain 5-HT levels following exercise (Zhang et al., 2020) that is required for exercise-induced adult hippocampal neurogenesis (Klempin et al., 2013). In the present study, we did not detect an increase in 5-HT levels in the hippocampus after exercise. This may be because 5-HT levels increased transiently, but not consistently, after exercise. However, we found that hippocampal 5-HT3R expression increased after exercise, which would enhance the efficiency of 5-HT3R activation by 5-HT.

This study has several limitations. We found that 5-HT3Rs were required for exercise-induced promotion of hippocampal neurogenesis; however, whether this enhanced neurogenesis increased synapse number and spine density, thereby facilitating hippocampal LTP, remains unclear and should be explored in future studies. Moreover, to understand more detailed mechanisms of the role of 5-HT3Rs in exercise-induced facilitation of LTP in the CA3 region, further investigation in mice with region-specific conditional knockout of 5-HT3R should be performed.

In conclusion, this study provides evidence for the critical role of 5-HT3Rs in exercise-induced improvement in spatial and exploratory memory. Our findings revealed a novel mechanism by which exercise promotes hippocampal CA3 LTP and enhances memory by up-regulating hippocampal 5-HT3R expression, thereby enhancing DG neurogenesis, astrocyte proliferation, and BDNF release in the hippocampus. More interestingly, we found that 5-HT3Rs are essential for exercise-induced enhancement of LTP in the CA3 region, but not in the CA1 or DG regions, of the hippocampus. This is the first report of heterogeneity in hippocampal LTP changes induced by exercise in subregions with different levels of 5-HT3Rs dependence. Considering the beneficial effects of exercise in patients with neurodegenerative disease, the 5-HT3R is a potential treatment target that should be investigated further.

## Additional files:

***Additional Figure 1:*** *Study flowchart.*

Additional Figure 1 Study flowchart.
5-HT3R: 5-Hydroxytryptamine type 3 receptor; E: exercise; KO: knockout; WT: wild-type.

***Additional Figure 2:*** *Experimental timeline.*

Additional Figure 2 Experimental timeline.
5-HT3R: 5-Hydroxytryptamine type 3 receptor; BrdU: 5-bromo-2'-deoxyuridine; D: day; E: exercise; HPLC: high-pressure liquid chromatography; LTP: long-term potentiation; MWM: Morris water maze; NOR: novel object recognition; TEM: transmission electron microscopy.

## Data Availability

*All relevant data are within the paper and its Additional files.*
